# Microsaccades during reading

**DOI:** 10.1371/journal.pone.0185180

**Published:** 2017-09-21

**Authors:** Norick R. Bowers, Martina Poletti

**Affiliations:** 1 Vision Science, University of California, Berkeley, CA, United States of America; 2 Department of Psychological and Brain Sciences, Boston University, Boston, MA, United States of America; Monash University, AUSTRALIA

## Abstract

Recent research has shown that microsaccades contribute to high acuity vision. However, little is known about whether microsaccades also play a role in daily activities, such as reading, that do not involve stimuli at the limit of spatial resolution. While the functions of larger saccades in reading have been extensively examined, microsaccades are commonly regarded as oculomotor noise in this context. We used high-resolution eyetracking and precise gaze localization to investigate fine oculomotor behavior during reading. Our findings show that microsaccade characteristics differ from those measured during sustained fixation: microsaccades are larger in size and primarily leftwards during reading, *i.e*. they move the line of sight backward on the text. Analysis of how microsaccades shift gaze relative to the text suggests that these movements serve two important functions: (1) a corrective function, by moving the gaze regressively within longer words when the preceding saccade lands too far toward the end of these words, and (2) an exploratory function, by shifting the gaze on adjacent words to gain additional information before the execution of the next saccade. Thus, microsaccades may benefit reading by enhancing the visibility of nearby words. This study highlights the importance of examining fine oculomotor behavior in reading, and calls for further research to investigate the possible roles of microsaccades in reading difficulties.

## Introduction

It is well established that analysis of eye movements during reading provides insights on the syntactic and semantic processing of sentences [1]. Most of the oculomotor research on reading has so far focused on the control of saccades larger than half a degree. In contrast, little attention has been paid to the possible functions of very small saccades (microsaccades). This has been a consequence of both the technical difficulty inherent in studying small eye movements, and the common view that microsaccades are too small to be beneficial in normal reading conditions [2–4]. Furthermore, previous research that looked at microsaccades during reading reported them to be rare [2, 3]. As a result, microsaccades have been often discarded as oculomotor noise in the reading literature [1].

Yet, recent studies have shown that microsaccades are important for high-acuity vision [5], and are finely tuned based on the task performed [5, 6]. Microsaccades are controlled similarly to large saccades [7–9], and their production appears to be mediated by the same neural structures [10, 11]. Moreover, these small eye movements have been shown to indicate the peripheral allocation of attention [12, 13]. These considerations call for a reassessment of microsaccade characteristics during reading.

When examining microsaccades it is crucial to keep in mind that different studies have used very different amplitude thresholds for defining these movements, ranging from 0.2° [2], to 2° [14] (see [15–17] on this issue). In fact, setting a threshold to define these eye movements is arbitrary given that saccade amplitude distributions are unimodal. Here we adopt a definition based on anatomical and physiological considerations, and define microsaccades as the saccades smaller than the radius of the foveola (<0.5°), the rod and capillary-free high acuity region of the fovea [18, 19]. Saccades in this amplitude range maintain the fixated stimulus within the foveola, yielding more than 50% overlap in the pre and the post-saccadic retinal input.

It is known that the amplitude of saccades scales with the size of the characters in the text [20]. These findings suggest that the range of text size for which microsaccades may be used similarly to saccades is too small to be functional for effective reading as more than two words should fit in 1°. However, it is possible that microsaccades play a role complementary to that of saccades with normal size text, or with text sizes slightly smaller than the most effective range for reading (*i.e*., character size <0.2°) [21]. Even if microsaccades shift the gaze over only a few characters in the text, they might still contribute to improve the discernibility not only of nearby, but also of more peripheral characters thanks to the shifts in attention with which they have been associated [22].

To investigate these possibilities, here we examined how microsaccades shift the line of sight with respect to the text. This is a challenging problem because of the difficulty in precisely localizing the center of gaze. Even with high-resolution eyetracking techniques, the error in gaze localization can be as large as 1 deg^2^ [23], an error too large to reliably examine where small eye movements shift the gaze on the text. To achieve high accuracy in gaze localization and to examine fine oculomotor behavior during reading, we relied on a high-precision Dual Purkinje Image (DPI) eyetracker [24, 25], and a state-of-the-art gaze-contingent calibration procedure that we recently developed [17, 26].

## Materials and methods

### Subjects

Ten emmetropic native English speakers (6 females, 4 males, average age: 23) took part in the experiment. A written informed consent was obtained from all participants following the procedures approved by the Boston University Charles River Campus Institutional Review Board. Participants were recruited using on-campus ads. All the recruited participants completed the study.

### Apparatus

The experiment was conducted in a dimly illuminated room. Stimuli were displayed on a fast phosphor monitor (Iyamaya HM204DT) at a resolution of 1024×768 pixels and vertical refresh rate of 85 Hz. Subjects were kept at a fixed distance of 123 cm from the monitor. A dental imprint bite bar and a head rest prevented movements of the head. The movements of the right eye were measured by means of a Generation 6 Dual Purkinje Image (DPI) eyetracker (Fourward Technologies). The internal noise of this system is ∼20 arcsec [17, 24], enabling a resolution of eye movements of approximately 1′ (1′ = 1/60^th^ of a degree) [25]. Vertical and horizontal eye positions were sampled at 1 kHz and recorded for subsequent analysis. Stimuli were observed monocularly, with the left eye patched, and were rendered by means of EyeRIS, a hardware/software system for gaze contingent display control that enables precise synchronization between eye movement data and the refresh of the image on the monitor [26].

### Task and procedure

**Fig 1:**
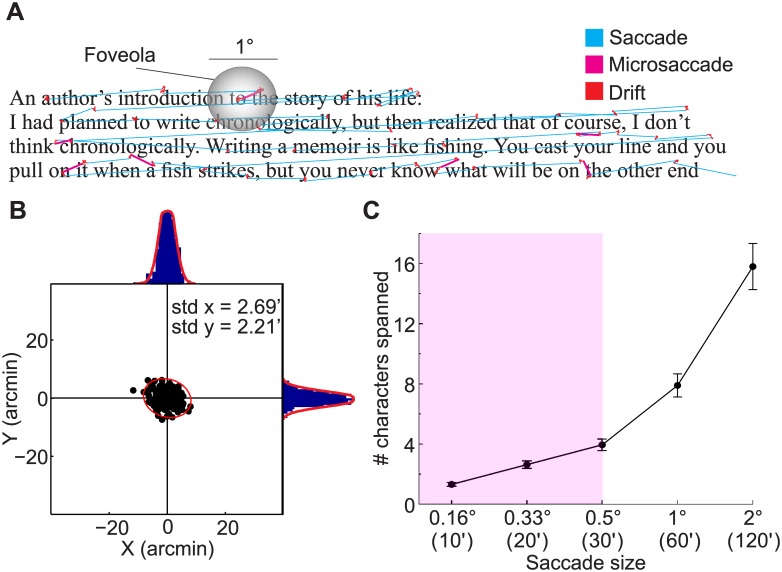
Methods and stimuli. (*A*) An example of eye movements during reading. The lines of text are an excerpt from one of the paragraphs used in the study. The size of the foveola (the high-acuity region of the retina) is also shown for comparison. Different types of eye movements are color-coded. (*B*) Accuracy in gaze localization. Error in gaze localization for one subject, each dot represents the offset between the gaze position and the fixated marker in an individual repetition of the calibration procedure (see Methods for details). The 99% confidence ellipse and the marginal distributions with their Gaussian fits are also shown. The numbers on the graph indicate horizontal and vertical standard deviations of the distribution. (*C*) Number of characters spanned as a function of saccade size. The shaded area in the graph represents the microsaccade range. Data points are averages across observers, and error bars represent s.e.m.

Stimuli consisted of passages of tenth-grade texts of various content printed in Times New Roman font (Fig 1*A*). The number of lines varied across passages (min: 3, max: 15, mean: 9.5), and an average of 12.7 ± 1.6 words were present in each line. The average length of words was 42′ ± 25′. Each passage was 10.6° wide along the horizontal dimension with the vertical dimension varying depending on the number of lines. The text was viewed at a distance of 123 cm and the X-height was equivalent to 9.6′ (0.16°). Participants were instructed to read each passage at their own pace and press a button on a joypad once they finished. After reading each passage, subjects answered a two-choice question regarding its content. The goal of the two-choice questions was to engage participants in the task, and to determine whether they paid attention to the content of the passages. The questions were easy if the paragraphs were read carefully, and subjects responded correctly to all questions. Passages were interleaved with a 5 s fixation trial during which steady fixation was maintained on a central marker.

### Calibration

Every session started with preliminary setup operations that lasted a few minutes. The subject was positioned optimally and comfortably in the apparatus. Subsequently, a calibration procedure was performed in two phases. In the first phase, subjects sequentially fixated on each of the nine points of a 3×3 grid, as it is customary in oculomotor experiments. These points were located 4.4° apart on the horizontal and vertical axes. In the second phase, subjects confirmed or refined the voltage-to-pixel mapping given by the automatic calibration. In this phase, they fixated again on each of the nine points of the grid while the location of the line of sight estimated on the basis of the automatic calibration was displayed in real time on the screen. Subjects used a joypad to correct the predicted gaze location, if necessary. These corrections were then incorporated into the voltage-to-pixel transformation. This dual-step calibration allows a more accurate localization of gaze position than standard single-step procedures, improving 2D localization of the line of sight by approximately one order of magnitude [5, 17]. A one-point gaze-contingent re-calibration procedure was performed before each trial to correct for possible small offsets caused by minute adjustments in head position.

To assess the localization accuracy of our approach, gaze position was measured at each point of the calibration grid during multiple repetitions of the calibration routine. The localization error estimated as the dispersion of the offset between the line of sight and the fixated point was less than 3′ on each axis (Fig 1*B*).

### Data analysis

Recorded eye movement traces were segmented into separate periods of drift and saccades. Classification of eye movements was performed automatically and then validated by trained lab personnel with extensive experience in classifying eye movements. Periods of blinks were automatically detected by the DPI eyetracker and removed from data analysis. Only trials with optimal, uninterrupted tracking, in which the fourth Purkinje image was never eclipsed by the pupil margin, were selected for data analysis. Eye movements with minimal amplitude of 3′ and peak velocity higher than 3°/s were selected as saccadic events. Saccades with an amplitude of less than half a degree (30′) were defined as microsaccades. Consecutive events closer than 15 ms were merged together into a single saccade in order to automatically exclude post-saccadic overshoots [27, 28]. Saccade amplitude was defined as the vector connecting the point where the speed of the gaze shift grew greater than 3°/s (saccade onset) and the point where it became less than 3°/s (saccade offset). Periods that were not classified as saccades or blinks were labeled as drifts. All data will be made available upon reasonable request.

Microsaccades were classified into three types based on their direction: vertical (either downward or upward, 45°-135° and 225°-315°, respectively, angles were measured counterclockwise relative to the horizontal axis), progressive (rightward microsaccades, <45° and >315°), and regressive (leftward microsaccades, >135° and <225°). Microsaccades were then further classified depending on whether they moved gaze across the written text, or whether they disrupted the normal text scanning pattern. Only the microsaccades landing less than 2.6′ away from the margins of the line of text being read were considered task-relevant, the others were classified as task-irrelevant. Task-irrelevant microsaccades disrupted the normal pattern of scanning the text, generally landing on the background or away from the line of text being scanned (*e.g*., most vertical microsaccades were classified as task-irrelevant). Task-relevant microsaccades were then separated into two main categories based on the onset and offset location of the microsaccade. We will use the term intra-word microsaccade to indicate microsaccades starting and landing on the same word, and the term inter-word microsaccade to indicate microsaccades that land on a different word. For example, a regressive intra-word microsaccade moves the gaze leftward on a single word, whereas a regressive inter-words microsaccade brings the gaze from one word to the preceding one. Spatial locations of the words on the display were obtained using the Optical Character Recognition toolbox in Matlab. The spatial borders of a word were defined as a rectangle encompassing all the characters in the word.

To determine whether the observed pattern of microsaccades was different from chance we used Monte Carlo simulations (*N* = 2,000). In one simulation the microsaccades performed during reading were replaced with microsaccades randomly chosen from those occurring during sustained fixation on a marker. While in another simulation microsaccades performed during sustained fixation were randomly placed over the text. The proportions of task-relevant and irrelevant microsaccades were calculated per each subject in each simulation and compared with the observed data.

## Results

We examined how microsaccades (here referred to as saccades smaller than 30′) relocate the line of sight with respect to the words in the text during reading of 10^th^ grade passages (Fig 1*A*). The size of the text was such that ∼ 8 characters fitted within the foveola. As shown in Fig 1*C*, microsaccades between 10′ to 30′ move the gaze over ∼ 1 to ∼ 4 characters, while larger saccades shift the gaze across up to ∼ 16 characters.

**Fig 2:**
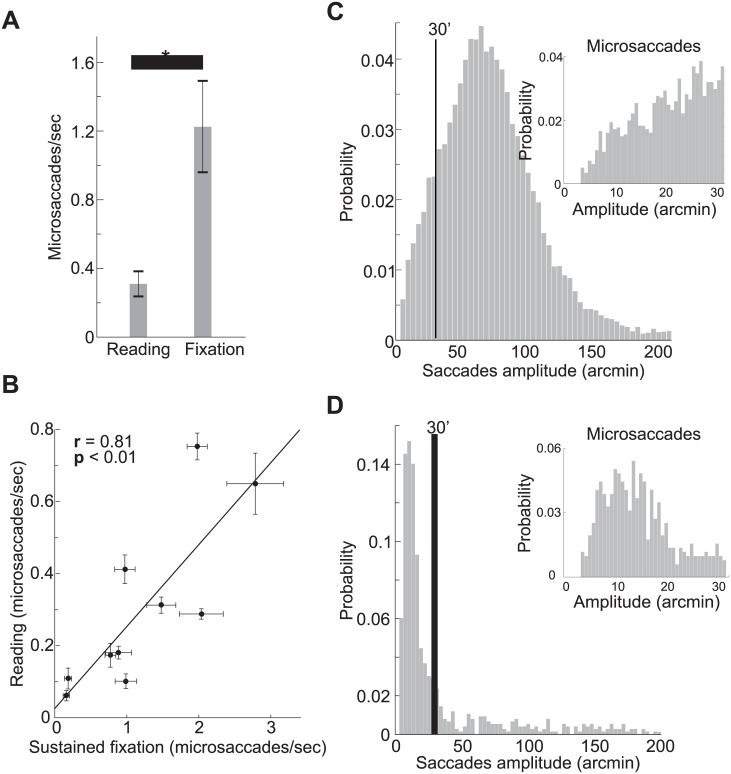
Microsaccades rates and amplitudes. (*A*) Rates of microsaccades during sustained fixation and during reading. The asterisk marks a statistically significant difference (*p*<0.01, two-tailed paired t-test). (*B*) Correlation between microsaccade rates in the two conditions. Each point represents the average rates for one subject. Error bars represent s.e.m. Probability distribution of saccade amplitudes during reading (*C*) and sustained fixation (*D*) across all the tested subjects. The vertical lines in the graphs mark the microsaccade threshold. The insets show microsaccade amplitude distributions.

Microsaccades occurred during reading in all the tested subjects (mean±std: 0.30±0.24 microsaccades per second); on average, a microsaccade was performed every 19.6±9.8 words. As illustrated in Fig 2*A*, the rate of microsaccades during reading was lower than the rate of microsaccades during sustained fixation, when the same subjects were simply asked to fixate on a marker at the center of the display (1.2±0.8 microsaccade per second, *p*<0.01, two-tailed paired t-test), the typical condition in which microsaccades are studied [29–31]. Yet, the rate of all saccades (both saccades smaller and larger than half a degree) was almost double in reading than during fixation (2.8 ± 0.3 saccades/s vs. 1.5 ± 0.7 saccades/s, *p*<0.0001, two-tail paired t-test).

Notably, inter-subjects differences were preserved across tasks (reading vs. sustained fixation, Pearson correlation coefficient *r* = 0.81, *p*<0.01, Fig 2*B*). That is, subjects characterized by lower/higher microsaccades rate during reading also showed lower/higher rates during sustained fixation. This finding suggests that each individual exhibits an idiosyncratic tendency to microsaccade, which is characterized by a specific level of microsaccades production, modulated in the same way across subjects on the basis of the task performed. Although the microsaccade rate is higher during sustained fixation, microsaccades do occur during reading: an average subject produces roughly 270 microsaccades in fifteen minutes, naturally raising the question of what function these movements may serve in this context.

**Fig 3:**
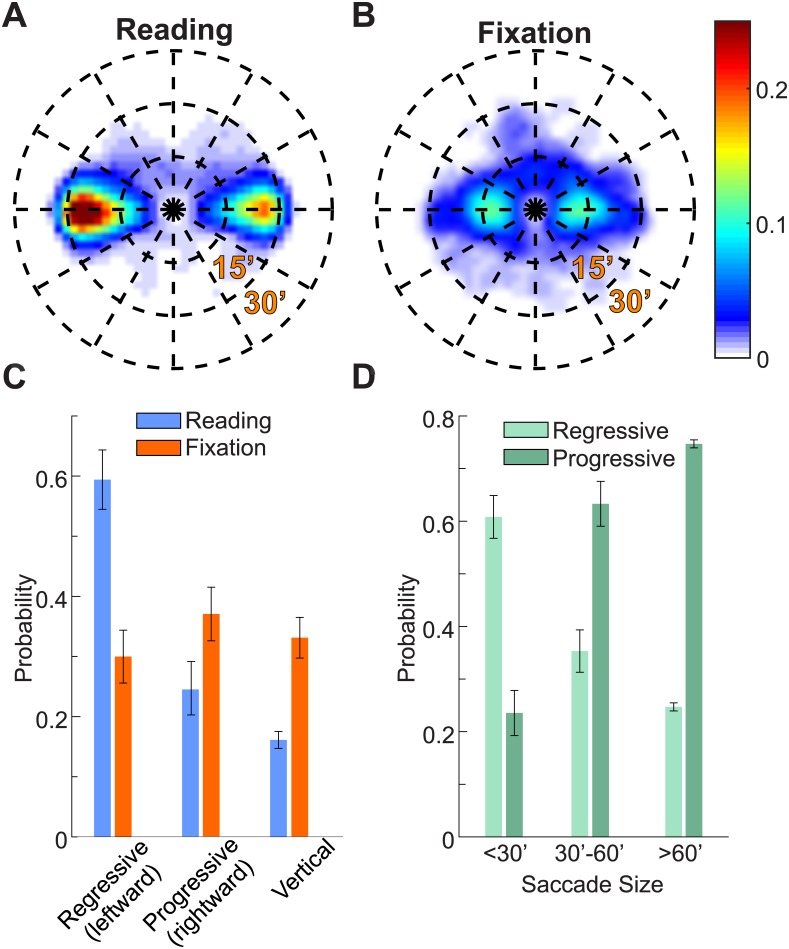
Microsaccades and saccades direction. Average 2D polar histograms of microsaccades direction and amplitude during reading (*A*) and sustained fixation (*B*). (*C*) Average probability of microsaccade direction during reading and during sustained fixation. Microsaccade direction was classified into three different categories; progressive (rightward), regressive (leftward) and vertical (up and downward). (*D*) Average probability of regressive and progressive saccades during reading as a function of saccade amplitude. Error bars represent s.e.m.

Amplitude and direction of microsaccades also differed between reading and sustained fixation. The amplitude of microsaccades was larger during reading (20′±1′ vs. 15′±4′, *p*<0.01, two-tailed paired t-test; see insets in Figs 2*C* and 2*D* and 3*A* and 3*B*). Moreover, as shown in Fig 3*C*, leftward (regressive with respect to the reading direction) microsaccade relocations were more frequent during reading (59% ± 16% vs. 30% ± 14%, ANOVA *F*(5,9) = 10.92, post-hoc Tukey-Kramer test, *p*<0.01). During fixation trials, aside from the known tendency to produce more horizontally oriented vs. vertically oriented microsaccades [31], there was no significant difference in the incidence of rightward, leftward and vertical microsaccades (post-hoc Tukey-Kramer test, *p*>0.9). Therefore, the pattern of microsaccades during reading differs considerably from the pattern of microsaccades during fixation.

**Fig 4:**
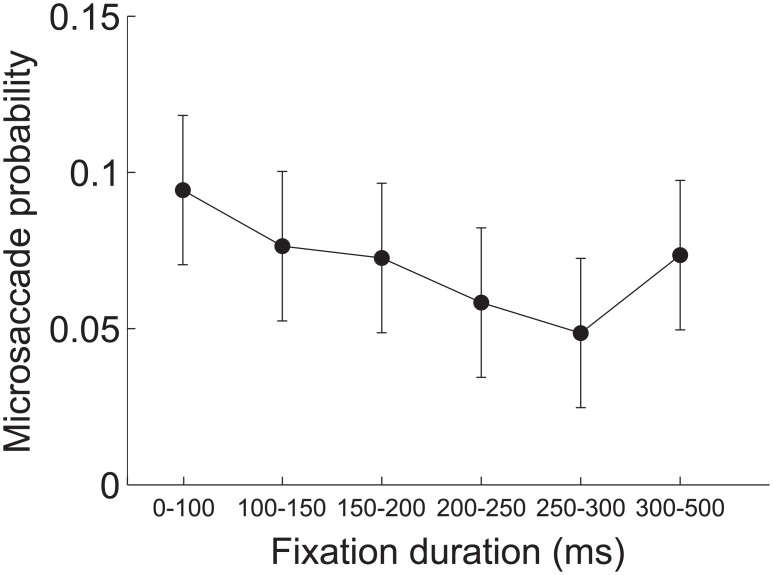
Microsaccades and fixation duration. Average probability of microsaccade occurrence as a function of the preceding fixation duration. Error bars represent 95% C.I.

It is possible that microsaccades are accidentally produced while reading; for example, when additional time is required to process a word subjects fixate longer and, as a consequence, a microsaccade is more likely to occur. To rule out this possibility we examined the duration of the fixation periods preceding saccades and microsaccades. On average, microsaccades were preceded by fixation periods of the same duration as those preceding saccades (222 ms ± 35 ms vs. 215 ms ± 29 ms; *p* = 0.48, two-tailed paired t-test). As illustrated in Fig 4, the probability of microsaccades occurrence did not change with the duration of the preceding fixation period (ANOVA *F*(5,9) = 1.8, post-hoc Tukey-Kramer test, *p*>0.05), and the duration of the preceding fixation did not change with the amplitude of saccades and microsaccades (ANOVA *F*(5,9) = 0.87, post-hoc Tukey-Kramer test, *p*>0.3). Therefore, the occurrence of microsaccades during reading was not related to the duration of the preceding fixation interval. Interestingly, however, slower readers were characterized by a higher rate of microsaccades; the slower the reading time, the higher the rate of microsaccades (Pearson correlation coefficient r = 0.85, p < 0.001).

We then analyzed the proportion of saccades moving the gaze either progressively or regressively on the words in the text during reading. As illustrated in Fig 3*D*, the ratio of progressive vs. regressive saccades increased with their amplitude. Saccades larger than 0.5 deg (2.9 ± 0.5 saccades/second) were primarily progressive (75%±2% progressive vs. 25%±2% regressive, *F*(5,9) = 35.18, post-hoc Tukey-Kramer test, *p*<0.01). In contrast, microsaccades were mostly regressive (61%±13% regressive vs. 24%±14% progressive; post-hoc Tukey-Kramer test, *p*<0.01). A similar tendency was previously reported by Kowler and Anton [3] using a different experimental paradigm.

**Fig 5:**
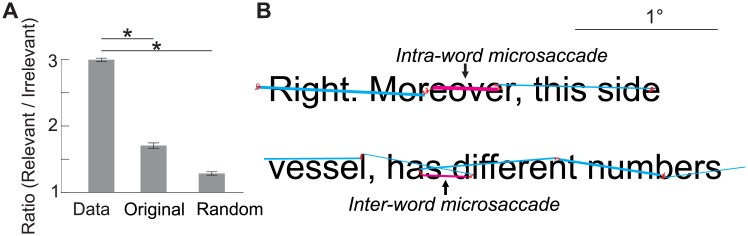
The pattern of microsaccades on the text. Microsaccades were divided into different categories depending on their starting and landing positions. (*A*) Average probabilities of task-relevant and task-irrelevant microsaccades obtained in three conditions: (1) the actual data, (2) by randomly placing the microsaccades performed during sustained fixation over the text (Random placement), and (3) by substituting the microsaccades performed during reading with those performed during sustained fixation (Original placement). Error bars represent s.e.m. Asterisks indicate statistically significant differences (*p*<0.01, two-tailed t-test). (*B*) Task-relevant microsaccades were mostly characterized by microsaccades branching two nearby words (Inter-word), and by microsaccades shifting the gaze within one word (Intra-word).

We further examined the pattern of microsaccades on the text based on their starting and landing positions. As illustrated in Fig 5*A*, task-relevant microsaccades, those with starting and landing position both on the current line of text (see Methods for details), were much more frequent (75%±2%) than task-irrelevant microsaccades (25%±2%, *p*<0.01, two-tailed paired t-test). Monte Carlo simulations were used to rule out the possibility that this difference was driven solely by the nature of the stimulus itself rather than being task-dependent, and to establish the ratio of relevant vs. irrelevant microsaccades in conditions in which microsaccades are produced independently from the text. In the first simulation we took the microsaccades performed during trials in which subjects maintained fixation on a marker, and placed them at random locations over the passages of text (random placement). In the second simulation (original placement) we replaced the microsaccades performed during reading with those performed during fixation on a marker. In this latter condition, the starting position of microsaccades on the text remained the same as in the original data but their landing position changed based on the amplitude and direction of the microsaccades performed during fixation. As illustrated in Fig 5*A*, the ratio of task relevant over task-irrelevant microsaccades was higher for the data collected during reading than the level obtained in both simulations (*p*<0.01, two-tailed paired t-test). These findings further support the idea that microsaccades are purposefully directed during reading.

To better understand the possible contributions of microsaccades we examined how task-relevant microsaccades relocated gaze on the words of the text in more detail. These microsaccades were classified into two main categories: “Intra-word” microsaccades, those that moved the gaze within a single word; and “Inter-words” microsaccades, those that shifted the gaze from one word onto a nearby word (see examples in Fig 5*B*). Our findings show that most task-relevant microsaccades relocated gaze on the same word (53%±7%). A smaller percentage of microsaccades branched between words (23%±6%). An even smaller percentage of task-relevant microsaccades served to either re-foveate the gaze back onto the text from the background (14%±6%), or to shift the gaze to a different line of text (10%±7%), which was then subsequently scanned by larger saccades.

**Fig 6:**
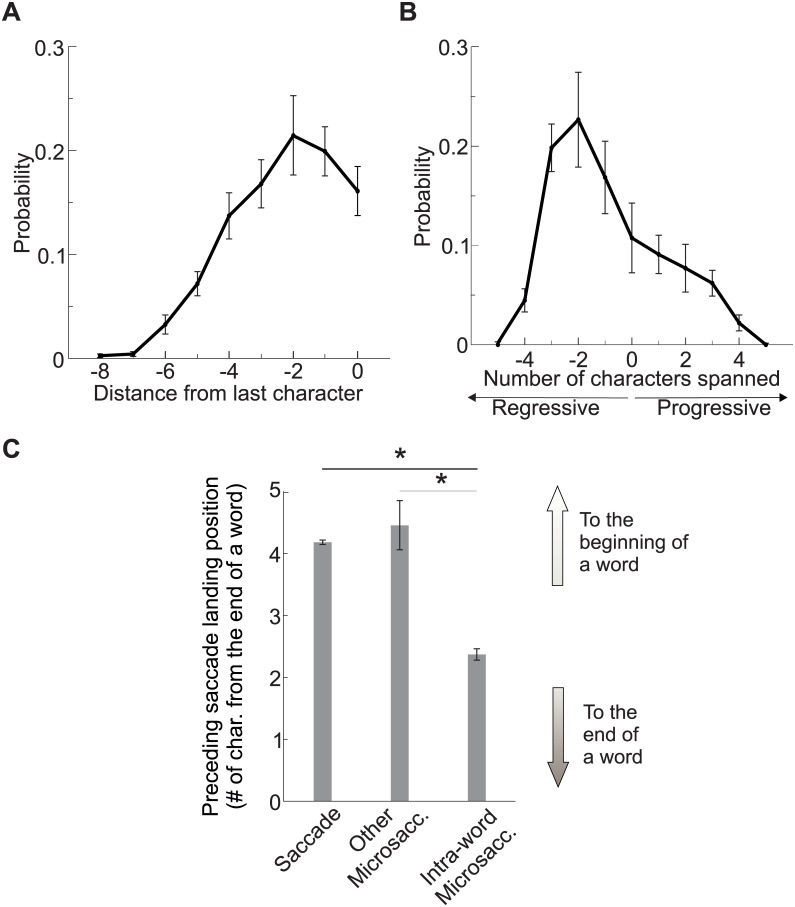
Intra-word microsaccadic gaze shifts. (*A*) Start position of intra-word microsaccades. The x-axis marks the number of characters from the last in a word. (*B*) Number of characters spanned by intra-word microsaccades. Positive and negative numbers indicate progressive and regressive shifts, respectively. (*C*) Landing positions of saccades followed by different types of eye movements (saccades, intra-word microsaccade and other microsaccades). Data in *C* refer to saccades landing on words longer than 7 characters. Error bars represent s.e.m.

Most intra-word and inter-word microsaccades were regressive. Intra-word microsaccades occurred most frequently on words longer than average (the average word length was 8.7 characters, ∼64′, vs. 4.9, ∼36′). As shown in Fig 6, intra-word microsaccades often started near the last character of a word (Fig 6*A*) and brought the eye 2 or 3 characters backward toward the center of the word (Fig 6*B*). These microsaccades presumably corrected for the preceding saccade; as illustrated in Fig 6*C*, the landing position of saccades preceding intra-word microsaccades was closer to the end of the word than the average landing position of all other saccades on words of comparable length. Thus, when a saccade landed too close to the end of a long word (> 7 characters), a microsaccade was likely to follow, bringing the eye back of few characters, an adjustment that appears to ensure optimal placement of the word within the foveola. These microsaccades, like those shifting gaze from the background to the text, appear to correct for saccades landing errors. In contrast, inter-words microsaccades and microsaccades shifting to a new line of text appear to serve a more exploratory function, in particular inter-words microsaccades may gather additional information on a word surroundings before planning the next saccade.

## Discussion

Our findings show that microsaccades are relatively frequent during reading, and that they are not simply the mere outcome of motor errors. The pattern of microsaccades during reading differs considerably from that occurring under sustained fixation. During reading microsaccades are predominantly leftwards and possess larger amplitudes. Whereas during sustained fixation microsaccades occur in all directions, and they cooperate with ocular drift to maintain the gaze on the target [32–34], during reading, microsaccades seem to play a role complementary to that of larger saccades.

Based on these findings we speculate that microsaccades during reading could possibly enhance visibility in two main ways: by correcting for saccadic landing errors and by moving the gaze, mostly regressively, over nearby words. Corrective microsaccades occur when saccades land toward the last few characters of a long word (> 7 characters); as high acuity vision starts to deteriorate already 10′ away from the preferred locus of fixation [5], landing at the end of a long word will cause part of its characters to fall away from the point of highest visual acuity. An intra-word regressive microsaccade can, therefore, optimally relocate the foveola on such long words allowing for a better visibility of all its characters. Importantly, microsaccades not only correct for saccades landing errors, they also enable exploration of nearby words. The word preceding the current fixation typically falls on a retinal region where vision is suboptimal, both because of retinal sampling and the perceptual span; the perceptual span during reading extends up to 14 letters to the right of the fixated location, but only a few letters to its left [35, 36]. If extra processing is needed, as for example, to eliminate semantic ambiguities, executing a microsaccade can be a very efficient strategy to acquire further information. Therefore, microsaccades may be particularly important for readers characterized by smaller perceptual spans, who typically tend to also be slower readers [37]. Consistent with this idea, our data show that reading times are positively correlated with the rate of microsaccades.

Our work represents a first step toward a better understanding of the role of microsaccades in reading. A natural question that arises based on our findings regards the actual impact of microsaccades on words visibility and comprehension. Future research should examine this issue, which can be addressed, for example, by having readers respond to a number of targeted questions on specific portions of the text, and evaluate their performance as a function of microsaccade occurrence on those sections of the text.

Importantly, microsaccades during reading are primarily regressive, moving the gaze backward on the text. Previous research linked the amount of regressive saccades to difficulties in reading [1, 38], and reported that regressive saccades normally represent only ∼ 10–15% of the total amount of produced saccades [39]. Here we show that these previous results do not extend to microsaccades. We report that regressive microsaccades represent the majority of microsaccades even for expert readers when reading simple text, indicating that they play an important role in normal reading and are not necessarily linked to reading difficulties.

While the perceptual span during reading is often treated as a static window that moves with the gaze, recent research [40] showed that it expands in the direction of the upcoming saccade, similarly to what happens during scene viewing [41, 42]. Therefore, microsaccades may be beneficial in reading not only by optimally relocating the stimuli within the foveola, but also by expanding the perceptual span, further enhancing visibility of nearby words. Moreover, the advantage of microsaccades likely extends to more peripheral words. It is known that microsaccades are associated with shifts of covert attention [12, 13], this is particularly important when reading as they may bring the benefits of a covert attentional shift toward peripheral words while maintaining the gaze on the word being read.

Our results highlight the importance of examining microsaccades during reading, and calls for further research investigating how microsaccades vary across individuals with different reading capabilities.
